# Camostat Mesylate Versus Lopinavir/Ritonavir in Hospitalized Patients With COVID-19—Results From a Randomized, Controlled, Open Label, Platform Trial (ACOVACT)

**DOI:** 10.3389/fphar.2022.870493

**Published:** 2022-07-22

**Authors:** M. Karolyi, E. Pawelka, S. Omid, F. Koenig, V. Kauer, B. Rumpf, W. Hoepler, A. Kuran, H. Laferl, T. Seitz, M. Traugott, V. Rathkolb, M. Mueller, A. Abrahamowicz, C. Schoergenhofer, M. Hecking, A. Assinger, C. Wenisch, M. Zeitlinger, B. Jilma, A. Zoufaly

**Affiliations:** ^1^ Department for Infectious Diseases and Tropical Medicine, Klinik Favoriten, Vienna, Austria; ^2^ Center for Medical Statistics, Informatics and Intelligent Systems, Medical University of Vienna, Vienna, Austria; ^3^ Department of Clinical Pharmacology, Medical University of Vienna, Vienna, Austria; ^4^ Department of Internal Medicine III, Clinical Division of Nephrology and Dialysis, Medical University of Vienna, Vienna, Austria; ^5^ Faculty of Medicine, Sigmund Freud University, Vienna, Austria; ^6^ Department of Vascular Biology and Thrombosis Research, Center of Physiology and Pharmacology, Medical University of Vienna, Vienna, Austria

**Keywords:** tmprss2, SARS-CoV-2, high-dose lopinavir/ritonavir, camostat mesylate, mortality, WHO scale, ACOVACT

## Abstract

**Background:** To date, no oral antiviral drug has proven to be beneficial in hospitalized patients with COVID-19.

**Methods:** In this randomized, controlled, open-label, platform trial, we randomly assigned patients ≥18 years hospitalized with COVID-19 pneumonia to receive either camostat mesylate (CM) (considered standard-of-care) or lopinavir/ritonavir (LPV/RTV). The primary endpoint was time to sustained clinical improvement (≥48 h) of at least one point on the 7-category WHO scale. Secondary endpoints included length of stay (LOS), need for mechanical ventilation (MV) or death, and 29-day mortality.

**Results:** 201 patients were included in the study (101 CM and 100 LPV/RTV) between 20 April 2020 and 14 May 2021. Mean age was 58.7 years, and 67% were male. The median time from symptom onset to randomization was 7 days (IQR 5–9). Patients in the CM group had a significantly shorter time to sustained clinical improvement (HR = 0.67, 95%-CI 0.49–0.90; 9 vs. 11 days, *p* = 0.008) and demonstrated less progression to MV or death [6/101 (5.9%) vs. 15/100 (15%), *p* = 0.036] and a shorter LOS (12 vs. 14 days, *p* = 0.023). A statistically nonsignificant trend toward a lower 29-day mortality in the CM group than the LPV/RTV group [2/101 (2%) vs. 7/100 (7%), *p* = 0.089] was observed.

**Conclusion:** In patients hospitalized for COVID-19, the use of CM was associated with shorter time to clinical improvement, reduced need for MV or death, and shorter LOS than the use of LPV/RTV. Furthermore, research is needed to confirm the efficacy of CM in larger placebo-controlled trials.

**Systematic Review Registration**: [https://clinicaltrials.gov/ct2/show/NCT04351724, https://www.clinicaltrialsregister.eu/ctr-search/trial/2020-001302-30/AT], identifier [NCT04351724, EUDRACT-NR: 2020–001302-30].

## Introduction

At the start of the SARS-CoV-2 pandemic, the antiviral effects of drugs such as hydroxychloroquine (HCQ), lopinavir/ritonavir (LPV/RTV), and ivermectin were evaluated as treatment options because they showed *in vitro* activity against SARS-CoV-2 and other coronaviruses (Caly et al., 2020; Choy et al., 2020; Liu et al., 2020; Sanders et al., 2020; Yao et al., 2020). Since none of these drugs improved time to clinical recovery or reduced 29-day mortality in randomized controlled trials, these drugs are considered obsolete for therapeutic use in COVID-19 (Cao et al., 2020; RECOVERY Collaborative Group, 2020; Amani et al., 2021; López-Medina et al., 2021; Popp et al., 2021; WHO Solidarity Trial Consortium et al., 2021). The two new oral antivirals molnupiravir and nirmatrelvir/ritonavir have been shown to reduce the rate of hospitalization in high-risk COVID-19 outpatients (Arribas et al., 2022; Caraco et al., 2022; Hammond et al., 2022; Jayk Bernal et al., 2022).

SARS-CoV-2 enters human cells by using its spike protein to bind to the ACE2 receptor, which is widely expressed in several human tissues, especially the respiratory tract (Hikmet et al., 2020; Sanders et al., 2020). Before entering the host cell, cleavage of the spike protein by human proteases such as the transmembrane protease serine 2 (TMPRSS2) is necessary (Hoffmann et al., 2020; Scudellari, 2021). Camostat mesylate (CM) and its metabolite 4-(4-guanidinobenzoyloxy) phenylacetic acid (GBPA) are inhibitors of TMPRSS2 and have shown *in vitro* efficacy against SARS-CoV-2 (Hoffmann et al., 2020; Breining et al., 2021; Hoffmann et al., 2021). This effect was confirmed in a small case series of patients critically ill with COVID-19 (Hofmann-Winkler et al., 2020). The drug is approved and widely used for chronic pancreatitis in Japan and has a very good safety profile (Talukdar et al., 2006; Breining et al., 2021). In a recently published randomized, controlled, double-blind trial of 205 hospitalized patients in Denmark, a dose of CM 200 mg three times daily (tid) for 5 days did not improve time to clinical recovery, disease progression, or mortality (Gunst et al., 2021).

In the Austrian Coronavirus Adaptive Clinical Trial (ACOVACT), a randomized, controlled, open-label, platform trial, we randomly assigned hospitalized patients with COVID-19 to receive either LPV/RTV or camostat mesylate, which was considered standard-of-care, for up to 10 days (NCT04351724, EUDRACT-NR: 2020–001302-30). The primary objective was to evaluate the efficacy of the study drugs on clinically relevant outcome parameters.

## Methods

### Intervention and Study Drugs

At the beginning of the trial, patients were randomized to one of three treatment arms: hydroxychloroquine (HCQ), with a loading dose of 200 mg two tablet bid and a maintenance dose of 200 mg one tablet bid; lopinavir/ritonavir (LPV/RTV) 200/50 mg two tablets two times daily (bid) (normal dose LPV/RTV); or camostat mesylate 100 mg two tablet bid. Treatment with camostat mesylate was considered standard-of-care. After the results of the SOLIDARITY and RECOVERY trials (RECOVERY Collaborative Group, 2020; WHO Solidarity Trial Consortium et al., 2021) were published, the study protocol was adapted. The HCQ arm was closed, and the LPV/RTV dosage was increased to a loading dose of 200/50 mg four tablet bid and a maintenance dose of three tablet bid (high-dose LPV/RTV). This decision was made based on both negative results using normal dose LPV/RTV and pharmacokinetic considerations.

After this amendment, patients were randomized in a 1:1 ratio to receive either high-dose LPV/RTV or camostat mesylate in the main study. If patients were randomized to receive LPV/RTV, concomitant medication was controlled interactions *via* the drug interaction checker of the University of Liverpool (https://www.covid19-druginteractions.org) and adapted if necessary.

### Other Treatment

All patients received low-molecular-weight-heparin in a prophylactic dose if no other indication for therapeutic anticoagulation (e.g., atrial fibrillation, history of pulmonary embolism, or deep vein thrombosis) or contraindication was present. Remdesivir was allowed if deemed necessary by the treating physicians. As soon as the findings of the RECOVERY trial were available, all patients requiring oxygen support received 6 mg dexamethasone for up to 10 days.

### Study Population and Randomization

This study was planned as a multi-center randomized, controlled, open-label, platform trial but conducted entirely at the 4^th^ medical department with infectious diseases and tropical medicine at the Clinic Favoriten in Vienna, Austria due to recruitment problems elsewhere. The first patient was enrolled on 20 April 2020, and the last patient was enrolled on 14 May 2021.

In the main study, we included hospitalized COVID-19 patients ≥18 years with laboratory-confirmed (i.e., PCR-based assay) infection with SARS-CoV-2 who required supplementary oxygen (due to oxygen saturation <94% on ambient air or >3% drop in case of chronic obstructive lung disease) or radiologically confirmed SARS-CoV-2 pneumonia. Women of childbearing potential needed to be willing to use effective contraceptive methods during the study. Exclusion criteria included severe liver dysfunction (e.g., ALT/AST >five times upper limit of normal), HIV infection or active viral hepatitis, anticipated discharge from hospital within 48 h, pregnancy or breastfeeding, and allergy or intolerances to the study medication. Furthermore, we excluded patients with an estimated life expectancy of <1 month (e.g., terminal cancer, etc.).

Each patient was randomized *via* an online tool which was provided by the medical university of Vienna and received a consecutive randomization number for the main study and a substudy if applicable. Enrollment and randomization were performed by the study team, which consisted of doctors in charge, doctors in training, or trained scientific staff. Patients, nurses, doctors, and the study team were aware of the treatment allocation in this open-label trial. All inclusion and exclusion criteria for the main and substudies and descriptions of the substudies are listed in the Supplementary Material.

Only the results of the main study (LPV/RTV vs. camostat mesylate) will be reported here.

### Primary and Secondary Outcome

The primary endpoint was time to clinical improvement which was defined as the time from randomization to a sustained improvement of at least one category on two consecutive days compared to the status at baseline. These measurements were performed using a seven-category ordinal scale. The seven categories of the World Health Organization’s proposed scale are as follows: 1) not hospitalized, no limitations on activities; 2) not hospitalized, limitation on activities; 3) hospitalized, not requiring supplemental oxygen; 4) hospitalized, requiring supplemental oxygen; 5) hospitalized, on noninvasive ventilation or high flow oxygen devices; 6) hospitalized, on invasive mechanical ventilation or ECMO; 7) death. The score was measured daily during hospitalization.

Secondary outcomes included time to sustained improvement of at least two categories compared to the status at baseline measured on the WHO ordinal scale, progression to mechanical ventilation or death, length of stay, and 29-day mortality.

### Ethics Approval

The study and all amendments during the study period were approved by the ethics committee of the Medical University of Vienna and Vienna ethics committee. Each study center defined its own standard-of-care treatment. Camostat mesylate was considered standard-of-care at the Clinic Favoriten where all patients were recruited. All methods were carried out in accordance with the ethical principles of the Declaration of Helsinki. All patients included in the trial signed an informed consent form.

### Statistical Analysis

A sample size calculation was performed based on the primary endpoint which was defined as time-to-clinical improvement for the comparisons of two groups. Based on Cao et al. (2020) publication (Cao et al., 2020), we assumed that the median time in treatment arm 2 (lopinavir/ritonavir) is about 16 days. Assuming an improvement between two groups of 6 days in the median time to clinical improvement, a log-rank test at a two-sided significance level of *α* = 0.05 with a sample size of 100 per treatment group would yield a power larger than 80%. These assumptions would translate to a hazard ratio of 1.6 when assuming exponential time-to-event curves.

The sample size calculation was performed using N-Query: when the sample size in each group is 100, with a total number of events required, E, of 154, an exponential maximum likelihood test of equality of survival curves with a 0.05 two-sided significance level will have 83.15% power to detect the difference between a Group 1 exponential parameter, λ₁, of 0.069 and a Group 2 exponential parameter, λ₂, of 0.043 (a constant hazard ratio of 1.6).

For qualitative variables (e.g., sex), absolute and relative frequencies will be calculated as per the treatment group. For quantitative data, mean ± standard deviation or median and the interquartile range are reported.

The primary endpoint time to sustained clinical improvement is visualized by Kaplan–Meier plots. Patients who died were censored on day 29 for this analysis (this corresponds to a Fine-Gray model with competing risks). The comparisons of the two treatment arms were performed using a log rank at a two-sided level alpha of 5%. Based on the Kaplan–Meier curves, median times with interquartile ranges are reported. Additionally, simple and multiple Cox-proportional hazard models were performed for the primary endpoint. In the Cox model treatment, arm was used as a factor. In the multiple Cox regression, we adjusted for additional baseline factors such as sex and age. The analyses of all secondary endpoints are considered exploratory, and no further correction for multiplicity was performed. For time-to-event endpoints, cumulative incidence curves and log-rank tests were calculated. Therefore, unadjusted *p*-values and, if appropriate, 95% confidence intervals are presented for secondary endpoints.

## Results

### Patients

Of the 210 randomized patients, 101 received CM and 100 received LPV/RTV, 96 of whom received high-dose LPV/RTV. Nine patients received HCQ and were not included in the analysis.

The mean age of the entire population was 58.6 years (SD 15.2), and 67% were male. The mean body-mass-index was 30.3 (SD 5.7). The three most common comorbidities were hypertension, diabetes mellitus, and chronic obstructive pulmonary disease.

Groups were similar with respect to all baseline characteristics.

The median time from symptom onset before treatment was 7 days (IQR 7–9) in both groups. The WHO score at baseline was comparable between the two groups, with 17% having a WHO score of 3 (20% LPV/RTV vs. 15% CM), 61% a WHO score of 4 (59% LPV/RTV vs. 63% CM), and 22% a WHO score of 5 (21% LPV/RTV vs. 22% CM). Thirty-three patients were treated with remdesivir. C-reactive protein, leukocyte, and lymphocyte count did not differ between groups. For further information, see Table 1.

**TABLE 1:** Baseline characteristics at randomization.

	LPV/RTV *n* = 100	Camostat *n* = 101	Total *n* = 201
Age (mean, SD)	60.7 years (12.6)	56.6 years (17.2)	58.6 years (15.2)
Sex			
Female	33 (33%)	34 (34%)	67 (33%)
Male	67 (67%)	67 (66%)	134 (67%)
BMI (mean, SD)	30.1 (5.7)	30.4 (5.6)	30.3 (5.7)
Symptoms before randomization (median, IQR)	7 days (4–9) *n* = 92	7 days (6–10) *n* = 90	7 days (5–9) *n* = 182
WHO Scale			
3 no oxygen	20 (20%)	15 (15%)	35 (17%)
4 low-flow oxygen	59 (59%)	64 (63%)	123 (61%)
5 high-flow oxygen/NIV	21 (21%)	22 (22%)	43 (22%)
Remdesivir	19 (19%)	14 (14%)	33 (16%)
Medical history			
Hypertension	58 (57%)	47 (47%)	105 (52%)
Diabetes mellitus	34 (34%)	20 (20%)	54 (27%)
Chronic obstructive lung disease	14 (14%)	16 (16%)	30 (15%)
Coronary artery disease	13 (13%)	14 (14%)	27 (13%)
Chronic kidney disease	8 (8%)	6 (6%)	14 (7%)
Congestive heart failure	5 (5%)	6 (6%)	11 (5%)
Cancer	3 (3%)	8 (8%)	11 (5%)
Laboratory parameters			
Leucocytes in G/l (mean, SD)	6.7 (2.9)	5.9 (2.4)	6.3 (2.7)
Lymphocytes in G/l (mean, SD)	0.98 (0.49)	0.96 (0.42)	0.97 (0.45)
CRP in mg/l (mean, SD)	79.5 (54.0)	72.1 (51.3)	75.7 (52.7)

SD, standard deviation; Md, median; IQR, interquartile range; BMI, body-mass-index; CRP, C-reactive protein.

### Primary Outcome

**FIGURE 1:**
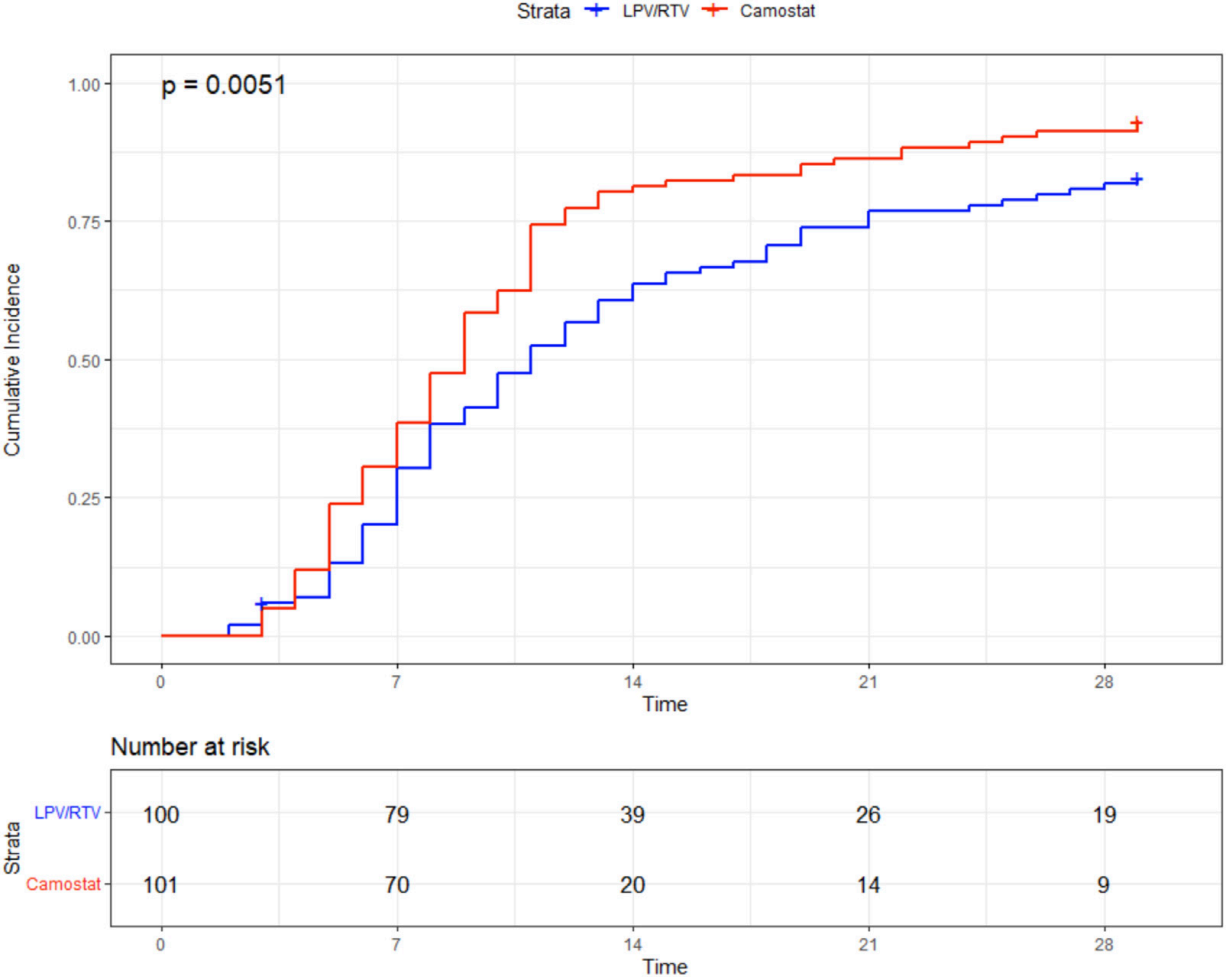
Primary endpoint: time (in days) to sustained (≥48 h) improvement ≥1 point in the WHO clinical progression scale.

**FIGURE 2:**
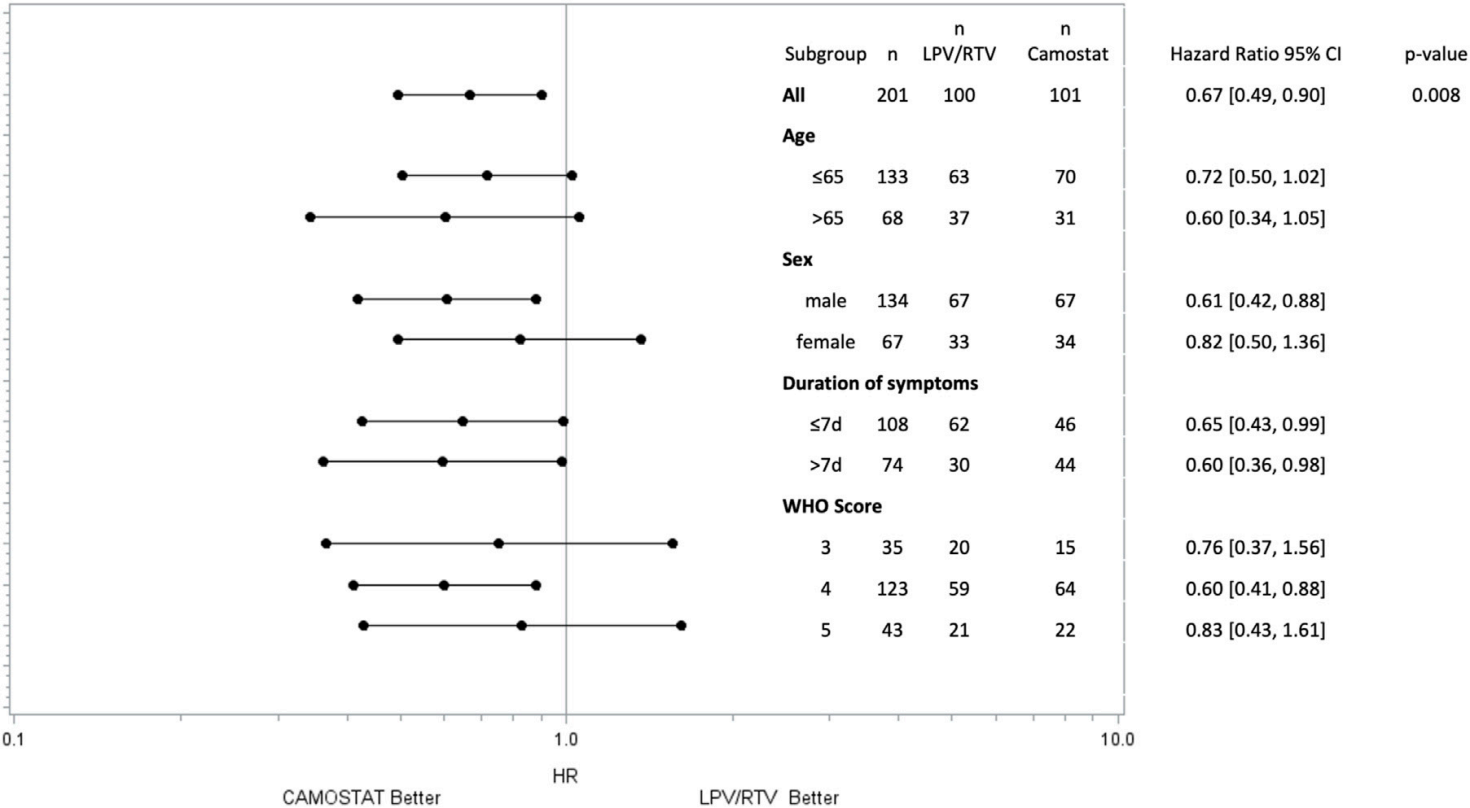
Time to sustained (≥48 h) improvement ≥1 point in the WHO scale by subgroups. *p*-values for subgroups are not displayed as the study was not powered for this analysis.

Median time to sustained clinical improvement (≥48 h) of at least one WHO scale category was 9 days (IQR 6–12) in the CM group and 11 days (IQR 7–21) in the LPV/RTV group (log rank test *p* = 0.005). The simple Cox regression model also showed a significant difference (HR = 0.67, 95%-CI 0.49–0.90, *p* = 0.008), see Figure 1 and Figure 2. In addition, sensitivity analysis in patients who received high-dose LPV/RTV vs. CM showed similar results (*p* = 0.005) (see Supplementary Figure S1).

The subgroup analysis by the WHO scale at the time of randomization showed a significant effect of CM on the primary outcome in patients with low-flow oxygen (*p* = 0.005) but not in patients without oxygen (*p* = 0.43) or on high-flow oxygen (*p* = 0.57), see Table 2. However, the trial was not powered to reveal subgroup effects.

**TABLE 2:** Outcome.

	LPV/RTV *n* = 100	Camostat *n* = 101	*p*-value
Primary outcome (Md, IQR)			
Overall	11 days (7–21)	9 days (6–12)	0.005
WHO 3 at baseline (*n* = 35)	17 days (9–27)	13 days (9–22)	0.43
WHO 4 at baseline (*n* = 123)	10 days (7–18)	7 days (5–11)	0.005
WHO 5 at baseline (*n* = 43)	11.5 days (6–20)	9 days (7–13)	0.57
Secondary outcome			
Time to improvement by ≥2 categories (Md, IQR)	13 days (9–28)	12 days (9–16)	0.021
Composite progression to MV or death*	15 (15%)	6 (5.9%)	0.036
Progression to MV*	13 (13%)	4 (4%)	0.023
29-day mortality	7 (7%)	2 (2%)	0.089
Length of stay (Md, IQR)	14 days (10–29)	12 days (10–19)	0.023

*For progression to MV patients who died without MV were censored at the time of death.

Md, median; IQR, interquartile range; MV, mechanical ventilation.

In the forest plot, further subgroup analyses for sex, symptom duration, and age are presented. Time of treatment initiation was not associated with the primary endpoint. In both subgroups (patients treated ≤7 days and >7 days after symptom onset), treatment with CM led to sustained clinical improvement more quickly than LPV/RTV (Figure 2).

### Secondary Outcome

When compared to patients in the LPV/RTV group, patients in the CM group had a significantly shorter time to clinical improvement within a 29-day period. This was demonstrated by improvement of at least two WHO scale categories (log rank test *p* = 0.021). The median time to clinical improvement was 12 days (IQR 9–16) in the CM group and 13 days (IQR 9–28) in the LPV/RTV group.

**FIGURE 3:**
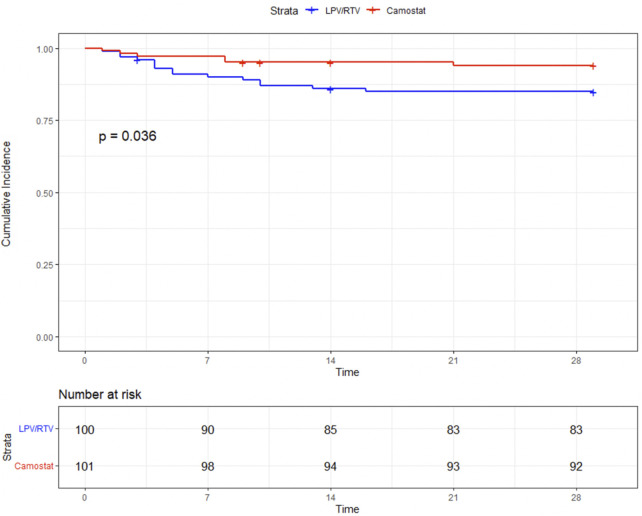
Time (in days) to mechanical ventilation or death.

The combined endpoint, time to progression to MV or death (whichever comes first within 29 days), occurred significantly less in the CM group [6/101 (5.9%) vs. 15/100 (15%), log-rank *p* = 0.036] (Figure 3). Thirteen patients received MV in the LPV/RTV group and four in the CM group. None of the mechanically ventilated patients treated with CM died within 29 days, whereas in the LPV/RTV group, five of 13 mechanically ventilated patients died within 29 days.

**FIGURE 4:**
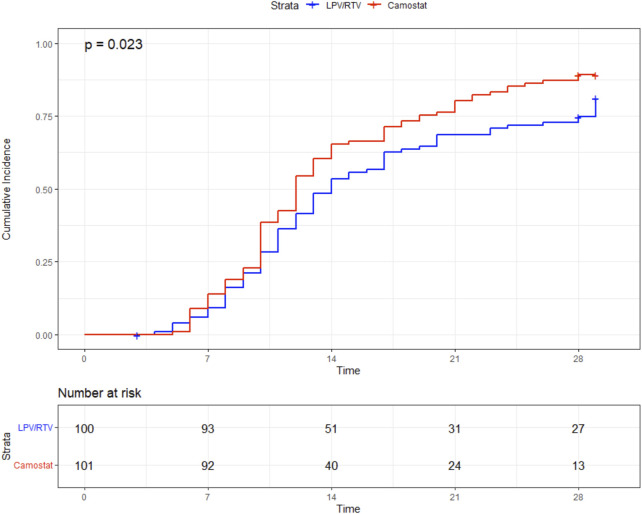
Time (in days) to discharge.

There was a statistically nonsignificant trend toward a lower 29-day mortality in the CM group than in the LPV/RTV group [2/101 (2%) vs. 7/100 (7%), log-rank *p* = 0.089]. In addition, patients in the CM group had a significantly shorter length of stay (12 days vs. 14 days, *p* = 0.023) (Figure 4). For further details, see Table 2.

### Safety

All adverse events with a potential relation to one of the study drugs have been reported and listed in Table 3. Overall, in the LPV/RTV group, significantly more patients experienced side effects (59% LPV/RTV vs. 36% CM, *p*-value 0.001). Severe side effects were rare (5% LPV/RTV vs. 2% CM). The most common side effects were elevated liver enzymes and diarrhea. Both were more frequent in the LPV/RTV group.

**TABLE 3:** Adverse events (AE).

	LPV/RTV *n* = 100	Camostat *n* = 101	*p*-value*
At least one AE	59 (59%)	36 (36%)	0.001
Nausea	8 (8%)	2 (2%)	
Diarrhea	21 (21%)	2 (2%)	
Abdominalgia	3 (3%)	1 (1%)	
Elevated liver enzymes	37 (37%)	23 (23%)	
Hyperbilirubinaemia	4 (4%)	1 (1%)	
Acute kidney injury	8 (8%)	5 (5%)	
Hypokalemia	4 (4%)	2 (2%)	
Hyperkalemia	2 (2%)	1 (1%)	
Cardiac arrhythmias	9 (9%)	7 (7%)	

*Fisher’s test, two sided.

## Discussion

In this randomized controlled trial (RCT), treatment with CM, which was considered standard-of-care, significantly reduced the time to sustained clinical improvement and the combined endpoint need for mechanical ventilation or death compared to LPV/RTV. There was also a trend toward lower 29-day mortality in patients treated with CM. The hospital stay for patients treated with CM was 2 days shorter.

To date, there has only been one RCT published that evaluated the effect of CM in hospitalized COVID-19 patients. This was performed in Denmark. In this trial, 205 patients were randomized in a 2:1 ratio to receive either CM (200 mg tid) or placebo for 5 days. Patients had a median age of 61 years, 60% were male, and the time from symptom onset to treatment initiation was 8 days. At baseline, approximately 30% did not require oxygen and 60% were on low-flow oxygen. During the trial, approximately 40% of the patients were treated with remdesivir and 60% with dexamethasone. No significant effect on time to clinical improvement, progression to ICU admission, or mortality was observed (Gunst et al., 2021). This is in contrast with the results of our trial which clearly showed a benefit regarding time to clinical improvement and in progression to mechanical ventilation or death in patients treated with CM. These different outcomes may, in part, be explained by the fact that there were several differences between the two trials. In our cohort, patients were treated for longer (up to 10 days versus 5 days) and tended to have a higher BMI. Additionally, treatment was started 1 day earlier after symptom onset and more patients needed high-flow oxygen at baseline.

Although our study was not powered for subgroup analysis, a benefit of CM in the subgroup of patients with low-flow oxygen could be shown. Despite the small sample size, the trend toward reaching the primary endpoint was consistent for all subgroups (see Figure 2).

A large retrospective observational trial which included 371 critically ill COVID-19 patients who were admitted to the ICU for treatment demonstrated significantly reduced in-hospital mortality (9.9% vs. 26.5%, *p* < 0.001) when CM (200 mg tid) was administered for 7 days. However, it is noteworthy that the study population was treated at the beginning of the pandemic, at a time at which treatment standards were different: only 20% received corticosteroids, approximately 40% were treated with tocilizumab, and almost every patient received favipiravir (Sakr et al., 2021). The observed trend toward a lower mortality rate in patients treated with CM in our study is consistent with this trial.

LPV/RTV neither improved time to clinical recovery nor did it reduce 29-day mortality in large RCTs. These trials administered the standard dose of LPV/RTV (200/50 mg of two tablets bid) which is commonly used in HIV patients (Cao et al., 2020; RECOVERY Collaborative Group, 2020; WHO Solidarity Trial Consortium et al., 2021). This standard dose does not lead to LPV plasma levels surpassing the EC_50_ of LPV for SARS-CoV-2 (Baldelli et al., 2020; Gregoire et al., 2020; Schoergenhofer et al., 2020), which might explain the lack of any clinical benefit with normal dose LPV/RTV. We adapted our study protocol shortly after study initiation, and a higher LPV/RTV dosage was used (loading dose of 200/50 mg four tablet bid and a maintenance dose of three tablet bid). However, this high-dose LPV/RTV regimen also failed to lead to LPV plasma levels above the EC_50_ of LPV for SARS-CoV-2, and the LPV steady-state plasma levels between the standard and high-dose did not differ significantly, which has been recently demonstrated by us (Karolyi et al., 2021). This suggests that even high-dose LPV/RTV does not have any meaningful SARS-CoV-2 antiviral effects, and this group can be considered the placebo group in our study.

Furthermore, to rule out possible toxic drug–drug interactions as a cause for a higher number of patients requiring mechanical ventilation in the LPV/RTV group, concomitant medication was evaluated prospectively in every patient. No potential interaction was found.

While significantly more side effects were reported in the LPV/RTV group, the number of adverse events was high in both groups. Most side effects are, however, typical symptoms and/or complications of COVID-19 and most likely not related to the study drugs. Diarrhea and elevated liver enzymes were the most common side effect and were reported more often in the LPV/RTV group. These are well-described side effects of LPV/RTV and therefore most likely drug-related. Severe side effects were rare but occurred more frequently in the LPV/RTV group. Overall, we observed no study drug-related adverse events in the CM group.

The strength of our study is that it is the first RCT that could demonstrate that CM, an oral bioavailable antiviral drug, has a positive effect on the time to clinical recovery in hospitalized patients with COVID-19. Sensitivity analyses showed that CM had the same effect on the primary outcome. CM has been used for decades in Japan, has a good safety profile, and can be administered easily. Newer oral antivirals such as molnupiravir and nirmatrelvir/ritonavir have only proven to be effective when treating outpatients with COVID-19 infections in RCTs (Arribas et al., 2022; Caraco et al., 2022; Hammond et al., 2022; Jayk Bernal et al., 2022). Furthermore, these agents are much more expensive than CM.

Our study has some limitations. It was a single-center open-label study which might reduce the generalizability of our results. Theoretically, the high dose of LPV/RTV may have led to a worse outcome in this group due to potentially toxic plasma levels. This, however, seems unlikely because in a separate analysis, we could show that the increased dose did not result in increased steady-state LPV/RTV plasma levels (Karolyi et al., 2021). Our study did not include a placebo group. As plasma levels of high dose LPV/RTV did not seem to differ significantly from those reached with a standard dose of LPV/RTV and were demonstrated to be too low to reach EC_50_ (Karolyi et al., 2021), we believe that the LPV/RTV can be seen as comparable to a placebo group. Additionally, several trials have shown that LPV/RTV does not have any effect on the outcome of patients with COVID-19 (RECOVERY Collaborative Group, 2020; WHO Solidarity Trial Consortium et al., 2021). While a thorough analysis of the adverse events in our study did not reveal any severe adverse events related to LPV/RTV we can, of course, not rule out that high-dose LPV/RTV may have had a negative impact on clinical outcomes. The oral bioavailability of CM is food-dependent (lower when taken with food) (Kitagawa et al., 2021). We did not instruct our patients to take the medication without food. This might have attenuated the effect of CM in our study. Last, the study was not powered to show any differences in mortality. Patients infected with Omicron variants were not included in the study. Cell entry of the Omicron BA.1 strain is less dependent on TMPRSS2 than older variants, and this may reduce the potential efficacy of CM in currently circulating strains (Meng et al., 2022).

In summary, the use of CM 200 mg bid for up to 10 days in hospitalized COVID-19 patients was associated with a shorter time to sustained clinical improvement, reduced need for MV or death, and a shorter length of stay compared to the use of LPV/RTV. These promising results make CM a potential candidate for treatment of patients hospitalized with COVID-19. Its low costs and oral formulation could simplify its widespread use.

Further research is needed to confirm the efficacy of CM in larger placebo-controlled trials and optimize the timing and dosage of CM as well as its effectiveness if concomitant treatment with other antiviral (e.g., remdesivir, molnupiravir, and nirmatrelvir/ritonavir) or immunosuppressive drugs (e.g. baricitinib, tocilizumab, and anakinra) is initiated.

## Data Availability

The raw data supporting the conclusions of this article will be made available by the authors, without undue reservation.
