# Supplementary material for: Camostat Mesylate Versus Lopinavir/Ritonavir in Hospitalized Patients With COVID-19—Results From a Randomized, Controlled, Open Label, Platform Trial (ACOVACT)
Source: Front Pharmacol. 2022 Jul 22;13:870493. doi: 10.3389/fphar.2022.870493 (PMC9354138; doi:10.3389/fphar.2022.870493)
Supplement: Supplementary file 1 [file Table1.DOCX]

**Supplementary Appendix**

Supplement to: Karolyi M, Pawelka E, Omid S, et al. Camostat mesylate versus lopinavir/ritonavir in hospitalized patients with COVID-19 patients – results from a randomized, controlled, open label, platform trial (ACOVACT).

This appendix has been provided by the authors to provide readers additional information.

# **Description of the substudies**

Inclusion in a substudy did not interfere with the administration of CM or LPV/RTV.

In substudy A (anticoagulation) patients were randomized to receive either rivaroxaban 5mg bid or standard of care, which was low-molecular-weight-heparin in a prophylactic dose administered subcutaneously once daily.

In substudy B (blood pressure) eligible patients were randomized to receive either the Renin-Angiotensin-System (RAS) blocking agent candesartan (starting dose 4mg once daily) or a non-RAS blocking agent (calcium channel blocker or alpha-blocker).

In substudy C patients were randomized to receive asunercept (25mg or 100mg or 400mg once weekly) or no additional drug.

# **Inclusion and exclusion criteria**

| **Inclusion criteria** | |
| --- | --- |
| Main study | Laboratory confirmed (i.e. PCR-based assay) infection with SARS-CoV-2 (ideally but not necessarily ≤72 hours before randomization for “antiviral” treatments) OR radiological signs of COVID-19 in chest X-ray or computed tomography* |
|  | Hospitalization due to SARS-CoV-2 infection (for anti-viral treatment arms) |
|  | Requirement of oxygen support (due to oxygen saturation 3% drop in case of chronic obstructive lung disease or confirmed SARS-CoV-2 pneumonia on chest X-ray. |
|  | Informed Consent obtained, the patient understands and agrees to comply with the planned study procedures, except for sub-study C: obtaining informed consent may be impossible due to the severe condition of the patient and may be waived |
|  | ≥18 years of age |
|  | For female patients with childbearing potential: willingness to perform effective measures of contraception during the study. |
| Sub-study A | eGFR of >20 mL/minSub-study A |
| Sub-study B | Outpatients with COVID-19 may be included |
|  | Blood pressure ≥135/85mmHG in 2 consecutive measurements OR patients with established and treated hypertension |
| Sub-study C | Signs of respiratory deterioration and progressing inflammation: need for oxygen supplementation, non-invasive ventilation, high-flow oxygen devices or mechanical ventilation AND CRP levels >5mg/dL (for Pentaglobin only), and admission to an ICU (for Pentaglobin only). |
| Qualitative study: | Fluent in German or English language |
| If for any given reason a patient does not qualify to participate in the main study, this will not preclude participation in sub-study C. | |
| *In case of negative PCR but clear radiological signs of COVID-19 patients have to be retested with serial nasopharyngeal swabs and PCR and, if possible antibody based assays. In any case, a laboratory based proof of COVID-19 is required or else the subject may be excluded from the per protocol analysis. | |

| **Exclusion criteria** | |
| --- | --- |
| Main study | Moribund or estimated life expectancy |
|  | Patient does not qualify for intensive care, based on local triage criteria |
|  | Pregnancy or breastfeeding |
|  | Severe liver dysfunction (e.g. ALT/AST > 5 times upper limit of normal) |
|  | Stage 4 chronic kidney disease or requiring dialysis for direct anticoagulant treatment |
|  | Allergy or intolerances to any of the experimental substances -> exclusion for the respective treatment arm; for asunercept known hereditary fructose intolerance |
|  | Anticipated discharge of hospital within 48 hours (for anti-viral treatment arms) |
| Contraindications treatment arm 2 (lopinavir/ritonavir) | severe hepatic impairment, CYP3A4/5 metabolized drugs as deemed relevant by treating physicians, HIV positive |
| Sub-study A | active bleeding or bleeding diathesis, lesion or condition considered as major risk factor for bleeding, recent brain or spinal injury, recent brain or spinal or ophthalmic surgery, recent intracranial hemorrhage, known or suspected esophageal varices, arteriovenous malformations, vascular aneurysms, major intraspinal or intracerebral vascular abnormalities. |
|  | ongoing therapeutic anticoagulation, which will continue, according to clinical practice |
| Sub-study B | chronic heart failure, allergies, hypersensitivities and intolerances, severe hepatic impairment and/or cholestasis, concomitant therapy with aliskiren-containing medications (for patients with diabetes mellitus or a GFR<60ml/min/1.73m^2^), known significant bilateral renal artery stenosis or renal artery stenosis of a solitary kidney |
| Sub-study C | Known active HIV or viral hepatitis |
| Asunercept | Females of childbearing potential |
| Qualitative study | participants who feel physically or emotionally unable to participate in the interviews or express their unwillingness at the time of study inclusion or scheduling interview appointments |

# **Sensitivity analysis**

Figure S1: Primary endpoint only considering high dose LPV/RTV vs. CM


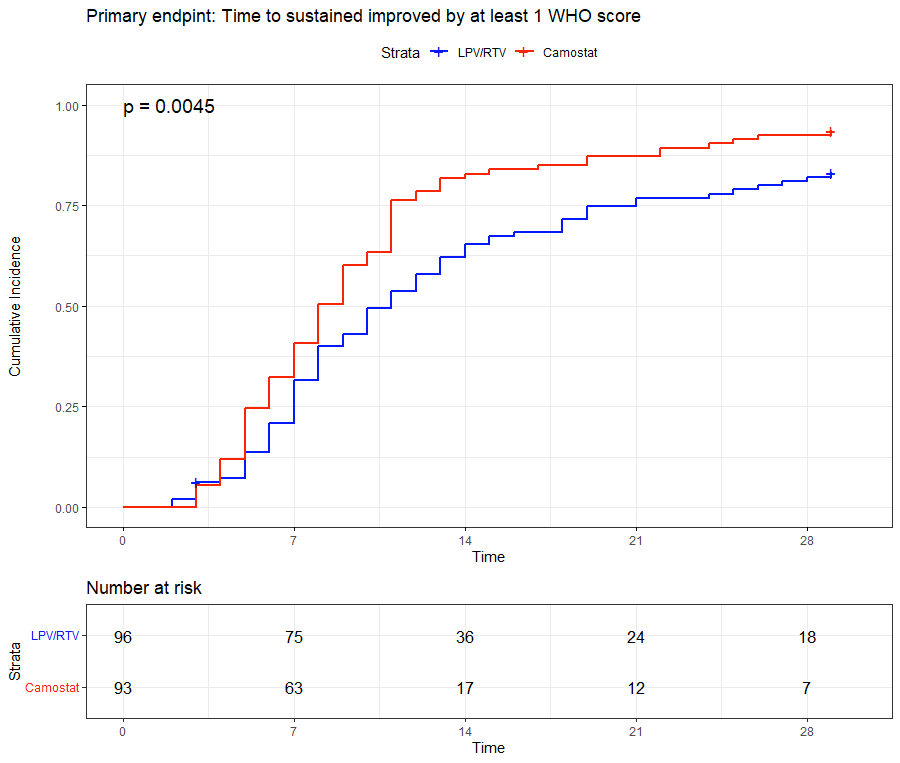


Figure S2: Study flow diagram

**Hydroxychloroquine n=9**

- study arm was closed due to results of other studies
- patients not included in the analysis

**Camostat mesylate (standard-of-care) n=101**

**Lopinavir/ritonavir (LPV/RTV) n=100**

- 4 patients received standard dose LPV/RTV
- 96 patients received high-dose LPV/RTV

**Randomized patients (N=210)**

- Enrollment and randomization were performed by trained medical staff via an online tool
